# Application of Molecular Topology for the Prediction of Reaction Yields and Anti-Inflammatory Activity of Heterocyclic Amidine Derivatives

**DOI:** 10.3390/ijms12021281

**Published:** 2011-02-22

**Authors:** Jordi Pla-Franco, María Gálvez-Llompart, Jorge Gálvez, Ramón García-Domenech

**Affiliations:** Molecular Connectivity and Drug Design Research Unit, Faculty of Pharmacy, Department of Physical Chemistry, University of Valencia Avd, V.A. Estellés, s/n 46100-Burjassot, Valencia, Spain; E-Mails: jorpla3@alumni.uv.es (J.P.-F.); galloma@alumni.uv.es (M.G.-L.); jorge.galvez@uv.es (J.G.)

**Keywords:** QSAR analysis, molecular topology, multilineal regression analysis, amidine derivatives, yield reaction, anti-inflammatory activity

## Abstract

Topological-mathematical models based on multiple linear regression analyses have been built to predict the reaction yields and the anti-inflammatory activity of a set of heterocylic amidine derivatives, synthesized under environmental friendly conditions, using microwave irradiation. Two models with three variables each were selected. The models were validated by cross-validation and randomization tests. The final outcome demonstrates a good agreement between the predicted and experimental results, confirming the robustness of the method. These models also enabled the screening of virtual libraries for new amidine derivatives predicted to show higher values of reaction yields and anti-inflammatory activity.

## 1. Introduction

Solvent-free reactions show a number of features that meet several of the principles of Green Chemistry, such as preventing formation of waste, increasing atom economy and savings in the use of auxiliary compounds [1]. In addition, these techniques can reduce the amount of hazardous products formed in chemical processes and increase the selectivity and yield of many organic reactions [2].

In this paper, we have focused on reactions employing microwave radiation. Microwave dielectric heating uses the capability of some solids or liquids to transform the electromagnetic energy into heat. Moreover, its magnitude depends on the properties of the molecules, so microwave radiation can be used to introduce a certain degree of selectivity [3] in the chemical process/es under study. Main applications of this technique are, among others, microwave assisted extraction [4], desorption and recovery of solids [5], SO_2_ emissions reduction and synthesis of organometallic [6], organic and inorganic compounds [7,8].

Furthermore, there are many approaches that have been proposed to ease the prediction of molecular properties. Equations linking quantitative structure–property (QSPR) relationships are particularly relevant and can be applied to large libraries of compounds for virtual computational screening [9,10]. However, these models require good structural descriptors that reliably represent the molecular features responsible for the property of interest.

Molecular Topology (MT) has largely demonstrated its efficacy in depicting molecular structures and predicting their properties. It follows a two-dimensional approach only considering the internal arrangement, including atoms. The structure of each molecule is represented by specific subsets of topological indices (TIs). These indices, when well chosen, provide a unique way of characterizing a molecular structure [11]. TIs are able to characterize the most important features of molecular structure: molecular size, binding and branching. The computation of TIs is very swift and they also have the advantage of behaving as true structural invariants. This means that TIs are independent of the spatial position of the atoms in a particular moment, although extensions of the TIs, taking account of the three-dimensional structure, have been also devised [12,13].

MT has demonstrated to be an excellent tool in the prediction of physicochemical [14] and biological properties [15] of structurally heterogeneous groups of compounds.

Most pain likely to be suffered in a lifetime is sensitive to anti-inflammatory (AI) drugs, for instance myalgia, artralgia, cephalalgia, neuralgia, dysmenorrheal and acute or chronic inflammatory processes. Furthermore, they are often useful in the unrest linked to viral and bacterial processes. They constitute the first level treatment of pain in the World Health Organization (WHO) strategy. All this accounts for their selection as our object of study in this paper.

Amidine derivatives are well known for their broad range of pharmaceutically relevant properties (anticancer [16–18], antimicrobial [19], antifungal [20], antibacterial [21] to mention just a few). However, they also exhibit anti-inflammatory activity (AA) [22,23], and this is why we have focused our attention on them.

The purpose of this work is to build up some predictive models for the reaction yield, and the anti-inflammatory activity, of a set of heterocyclic amidine derivatives synthesized under environmental friendly conditions using microwave irradiation. Later on, the models were applied to virtual screening libraries in order to search for new amidine derivatives with higher values of reaction yields and anti-inflammatory activity.

## 2. Materials and Methods

### 2.1. Data Sets and Studied Reactions

All compounds used in the present study were collected from recently published literature sources [24]. The data set used comprises series of heterocyclic amidine derivatives.

The *in vivo* anti-inflammatory activities expressed as percentage of carrageenan edema inhibition was used on albino rats of Charles Foster strain, by adopting the method of Winter *et al.* [25].

**Figure 1.**
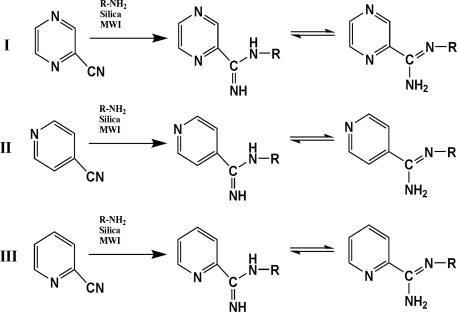
Synthesis of amidine derivatives.

The reaction studied in this work is the free-solvent synthesis described by Sondhi *et al.* [24] in one of his later works. In this study, several heterocyclic amidine derivatives were synthesized by condensation of 2-cyanopyrazine, 4-cyanopyridine and 2-cyanopyridine with furfurylamine, histamine, 1-(3-aminopropyl) imidazole, 4-picolylamine, 2-picolylamine, and tryptamine respectively, using microwave irradiation. The products obtained showed anti-inflammatory activity and achieved good reaction yields. Figure 1 shows reaction schemes while Table 1 shows the products and their respective properties.

### 2.2. Molecular Descriptors

Molecular descriptors used in the present work were topological indices (TI) which are described in Table 2, along with their definitions and references.

The chemical structure of each compound was drawn with the aid of the Chemdraw Software Package, Version 10. Each compound was characterized by a set of 434 TIs obtained with Dragon Software, Version 5.4 [30]. They were computed from the adjacency topological matrix obtained from the hydrogen depleted graph.

### 2.3. QSAR Algorithms: Multilinear Regression Analysis

The general purpose of multilinear regression analysis (MLRA) is to outline the relation between two or more independent variables and a dependent variable, by fitting a linear equation to observed data.

The regression equations were obtained by correlating the experimental yields values and the percentage of carrageenan edema inhibition of the 16 compounds from the database with the aforementioned TIs. We used the software package Statistica Version 8.0 to develope multilinear regression analysis, MLRA.

Variable selection was carried out by means of the Furnival–Wilson algorithm and variable sets with the minimal Mallows’ Cp were selected as optimal for the regression equations [31].

Typically, the quality and robustness of the model must be verified by using different types of validation criteria. In this article, as our data set was small, n = 16, we used the internal validation or cross-validation with a leave-one-out procedure (LOO) and a randomization test.

In the LOO algorithm, one case is eliminated from the data set and then the regression analysis, with the N-1 remaining cases and the original descriptors (the ones selected in the first regression), is performed again. The corresponding property value for the removed case is then predicted. This procedure is repeated as many times as there are cases in the data. The value of prediction coefficient, Q^2^, indicates the quality of the prediction function selected.

In the randomization test, the values of the property of each compound are randomly permuted and linearly correlated with the aforementioned descriptors.

### 2.4. Molecular Screening

Molecular topology is an efficient tool showing some advantages over other more well known approaches, such as molecular mechanics or quantum chemistry. The most remarkable advantage is perhaps the calculation speed. Hundreds of compounds can be analyzed within a few minutes time frame.

For this reason, molecular topology is well suited to evaluate possible biological activities of compounds represented in large databases or virtual libraries.

If the predictive power of the QSAR model obtained is satisfactory, it can be used to record and optimize the property analyzed.

In this paper, we have designed a library of heterocyclic amidine derivatives using the scheme reaction II illustrated in Figure 1, by using different substituents in the R position.

Only the compounds predicted to be active and with a high reaction yield were selected as potential interesting candidates.

## 3. Results and Discussion

Searching for equations capable to predict reaction yields (logYield) and anti-inflammatory activity (logAA) of the analyzed amidine derivatives, was the first objective. The best linear equations obtained, and their statistical parameters, were:

(1) $$\begin{array}{l} \text{logYield}=3.927+0.029\text{Pol}\text{−}0.316\text{ATS}8\text{v}\text{−}0.534\text{EEig}01\text{d}\\ \text{with}\;\text{N}=16,\;\text{r}=0.884,\;{\text{r}}^{2}=0.782,\;{\text{Q}}^{2}=0.667,\;\text{SEE}=0.0117,\;\text{F}=14.3,\;\text{p}=0.0002; \end{array}$$

(2) $$\begin{array}{l} \text{and},\;\text{logAA}=10.384\text{−}1.324\text{EEig}09\text{x}\text{−}4.193\text{EEig}06\text{r}+2.404\text{EEig}10\text{r}\\ \text{with}\;\text{N}=16,\;\text{r}=0.906,\;{\text{r}}^{2}=0.820,\;{\text{Q}}^{2}=0.629,\;\text{SEE}=0.0671,\;\text{F}=18.2,\;\text{p}=0.00009. \end{array}$$

The above values of 0.75 and 0.5 of r^2^ and Q^2^, respectively, in addition to the low values of SEE in both cases (less than 12% of the average values of the property) confirm the validity of the models from a predictive standpoint.

**Figure 2.**
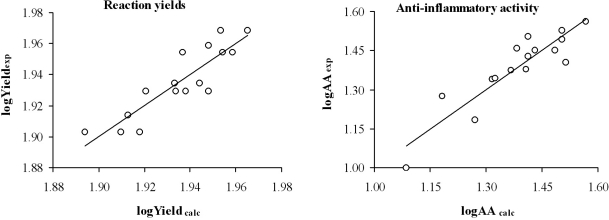
Graphic representation of logYield_exp_ *versus* logYield_calc_ and logAA_exp_ *versus* logAA_calc_ from the topological models selected.

Table 3 and Figure 2 show the yield and the anti-inflammatory activity predicted for each compound analyzed.

The EEig indices, topological descriptors derived from the eigenvalue of the adjacency matrix of edges weighed with different properties appear in both equations [29]. So, EEig01d takes into account the dipole moments of atoms, EEig09x the bond order of the various edges and EEig06r and EEig10r the resonance integral. Other indices present in Equation 1 are Pol, the number of polarity calculated as the number of pairs of vertexes at topological distance equal to 3 [26,27] and the Moreau-Broto autocorrelation index, ATS8v, weighed by Van der Waals volumes [28].

**Figure 3.**
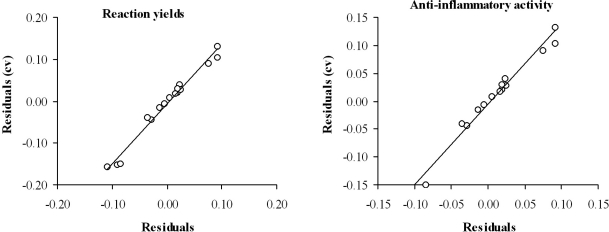
Graphic representation of cross-validated residuals *versus* residual for the topological models selected.

The predictive ability of the selected mathematical topological models was evaluated through cross-validation, using the leave-one-out test. Table 3 (columns 4 and 7) and Figure 3 show the obtained results. The values of Q^2^ = 0.667 for reaction yield and Q^2^ = 0.629 for anti-inflammatory activity are accepted as satisfactory [32].

**Figure 4.**
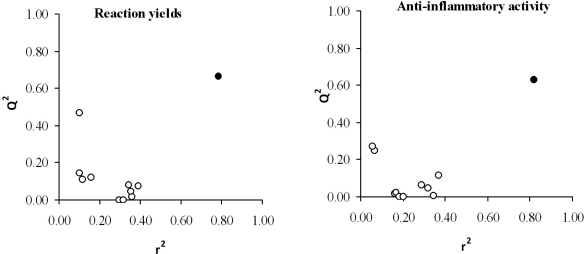
Graphic representation of the prediction coefficient, Q^2^, *versus* correlation coefficient, r^2^, obtained by randomization study with yield (left panel) and anti-inflammatory activity (right panel) shown. Black point: selected model.

In order to prevent the possible existence of fortuitous regressions, a *randomization test* was carried out. Thus, the values of the property of each compound are randomly permuted and linearly correlated with the aforementioned descriptors. This process is repeated as many times as needed. The usual way to represent the results of a randomization test is plotting the correlation coefficients *versus* the predicted ones, r^2^ and Q^2^, respectively. The results of the randomness tests, shown in Figure 4, suggest a high stability of both models (all regressions were rather poor except for the selected equation (black point) with the real values for each compound).

Once predictive equations were established, it was possible to carry out a search for new compounds showing anti-inflammatory activity, that could be reliably obtained from a highly efficient synthetic reaction. Based on the selected topological models, a virtual molecular screening, using the reaction scheme II and different structural fragments, was carried out. The results are exposed in Table 4. All proposed compounds, except 7a and 7c, show an expected yield exceeding 80%. With respect to the anti-inflammatory activity, compounds 7d–g exceeds the value of 50% in its pharmacological activity. In conclusion, it can be said that the proposed group of compounds is interesting from the anti-inflammatory activity standpoint.

Of course, these indicative results need to be confirmed by experimental tests. Should the test prove positive, the models proposed would be validated and could serve as a useful tool for the search of novel compounds synthesized under environmental friendly conditions and displaying anti-inflammatory activity.

## 4. Conclusions

Molecular topology was successfully used to arrange QSPR models for predicting the reaction yield and anti-inflammatory activity, in a group of 16 heterocyclic amidine derivatives, synthesized under environmental friendly conditions, using microwave irradiation. All the molecular descriptors used in this study were topological indices. The mathematical models achieved and described herein retain the main structural features of the correlatable properties, and hence can be applied to the search of new analogous compounds with an improved environmental profile.

## Figures and Tables

**Table 1:** Reaction yields and anti-inflammatory activity of amidine derivatives synthesized by microwave assisted methods. 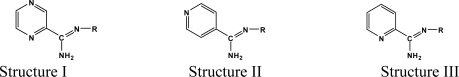

**Synthesis**	**Comp.**	**R**	**(%) Yield**	(%)AA*
I	3a	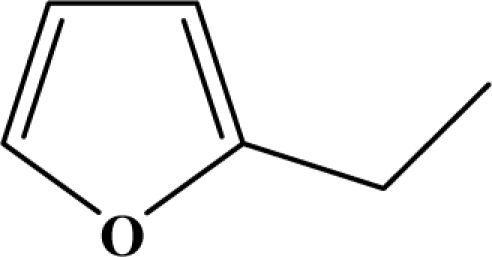	93	36.6
I	3b	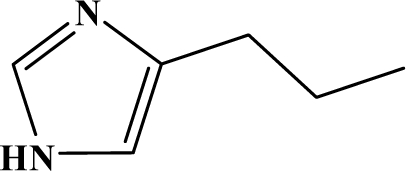	91	25.4
I	3c	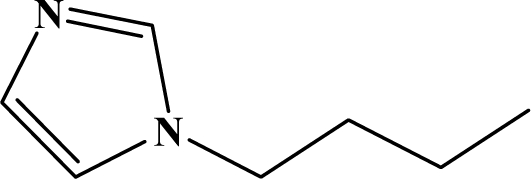	80	21.8
I	3d	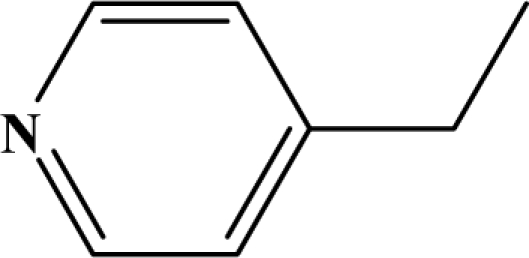	90	32.0
I	3f	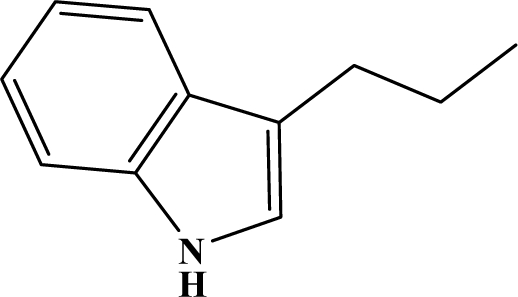	85	23.9
II	4a	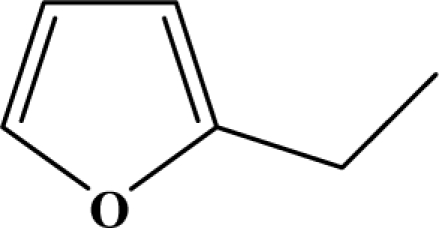	90	15.2
II	4b	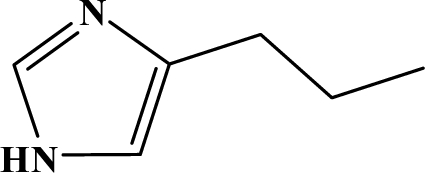	82	22.1
II	4c	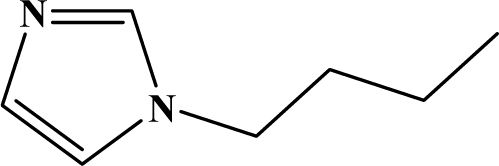	80	10
II	4d	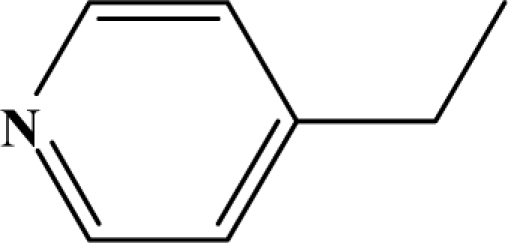	85	31.0
II	4e	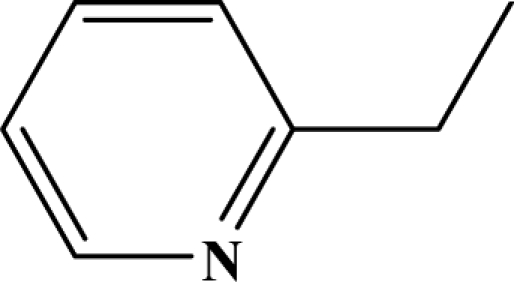	93	33.8
II	4f	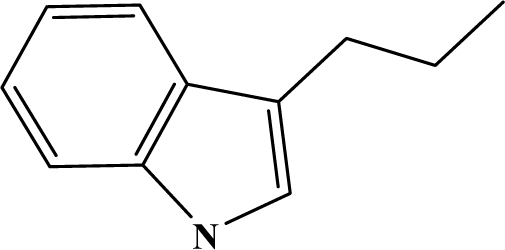	80	28.2
III	5a	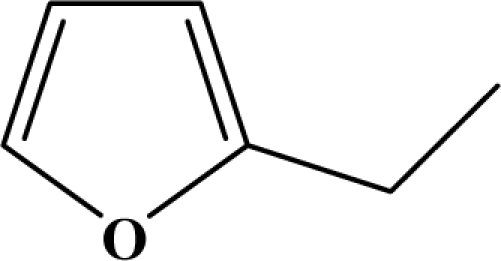	85	28.7
III	5b	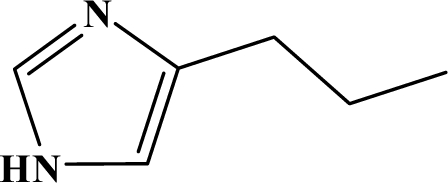	86	26.8
III	5c	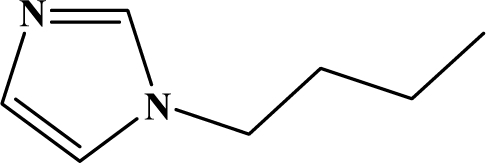	86	18.8
III	5d	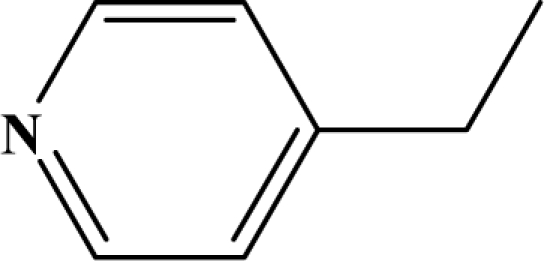	90	28.2
III	5f	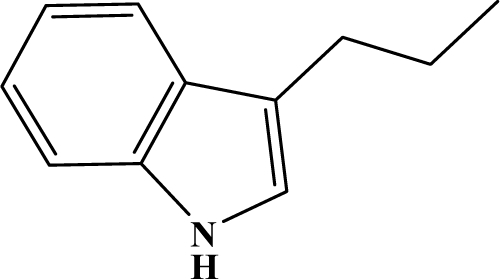	85	23.6

* Anti-inflammatory activity at a dose of 50 mg/Kg *p.o*. [24]. (Ibuprofen, reference drug with AA=39.0%).

**Table 2.** Descriptors used in this study.

**Symbol**	**Name**	**Definition**	**Refs.**
Pol	Polarity number	Number of pairs of vertexes at topological distance equal to 3	[26,27]
ATSkv k = 1–8	Moreau-Broto autocorrelation index of order k weighed by Van der Waals volumes	${\text{ATS}}_{\text{kv}}=\frac{1}{2}\sum_{\text{i}=1}^{\text{A}}\sum_{\text{j}=1}^{\text{A}}({\text{v}}_{\text{i}}\;{\text{v}}_{\text{j}}\delta ({\text{d}}_{\text{ij}}\;;\;\text{k}))\;$ v, Van der Waals volume □, Kronecker delta d_ij_, topological distance between i-atom and k-atom k, index order	[28]
EEigkd k = 1–15	K-st eigenvalue from weighted edge adjacency matrix	K-st eigenvalue from edge adjacency matrix weighted by dipole moments of atoms	[29]
EEigkr k = 1–15	K-st eigenvalue from weighted edge adjacency matrix	K-st eigenvalue from edge adjacency matrix weighted by the resonance integral	[29]

**Table 3.** Experimental and predicted values obtained for each compound analyzed through multilinear regression analysis.

**Comp.**	**Yield_exp_^a^ (%)**	**Yield_calc_^b^ (%)**	**Yield_calc_ (cv)^c^ (%)**	**AA_exp_^a^ (%)**	**AA_calc_^d^ (%)**	**AA_calc_ (cv)^e^ (%)**
3a	93	92	92	36.6	37.1	37.2
3b	91	89	88	25.4	32.6	36.4
3c	80	83	83	21.8	20.7	19.9
3d	90	91	91	32.0	25.9	25.2
3f	85	86	86	2.9	25.6	26.4
4a	90	90	90	15.2	18.7	21.6
4b	82	82	82	22.1	21.1	21.0
4c	80	78	78	10.0	12.2	14.1
4d	85	89	89	31.0	32.0	32.1
4e	93	90	89	33.8	32.0	31.7
4f	80	81	82	28.2	27.0	26.4
5a	85	87	87	28.7	24.1	23.3
5b	86	88	89	26.8	25.8	25.8
5c	86	86	86	18.8	15.2	13.9
5d	90	86	86	28.2	30.6	30.9
5f	85	83	83	23.6	23.3	23.2

a From reference [24];

b Calculated from Equation (1);

c From cross-validation with Equation (1);

d Calculated from Equation (2);

e From cross-validation with Equation (2).

**Table 4.** Computational screening applied to heterocyclic amidine derivatives and selection of theoretically anti-inflammatory compounds with high reaction yield. 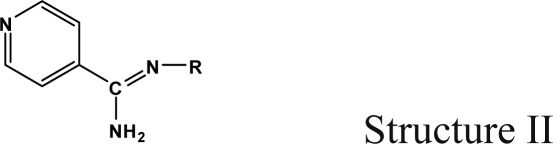

**Sinthesis**	**Comp.**	**R**	**Yield_calc_^a^ (%)**	**AA_calc_^b^ (%)**
II	6a	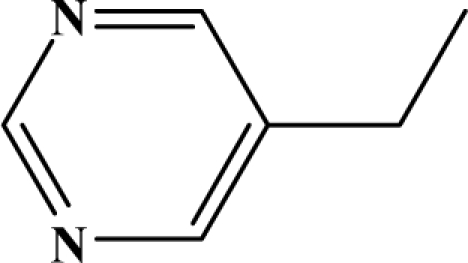	89.3	31.1
II	6b	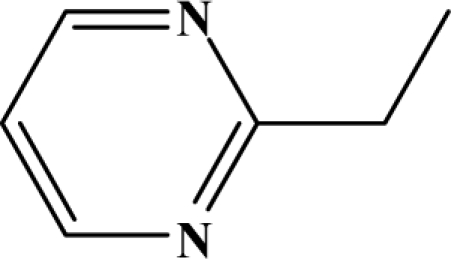	95.9	31.4
II	6c	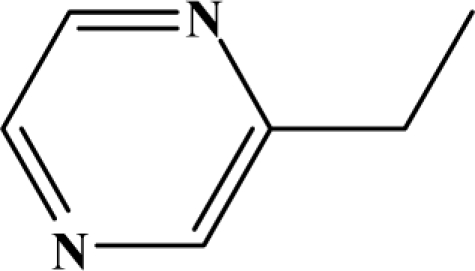	97.8	31.4
II	6d	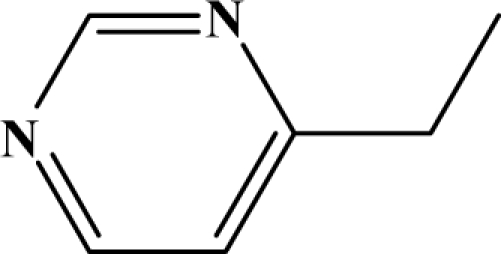	98.2	31.6
II	7a	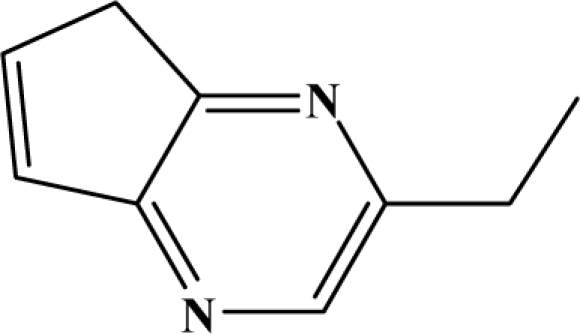	80.3	29.8
II	7b	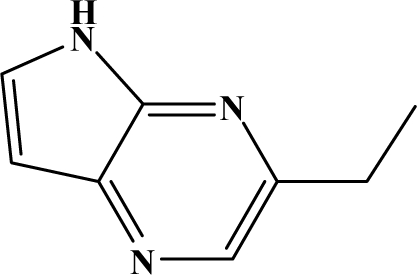	89.5	27.2
II	7c	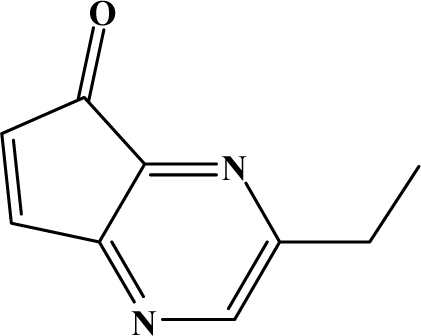	72.7	42.2
II	7d	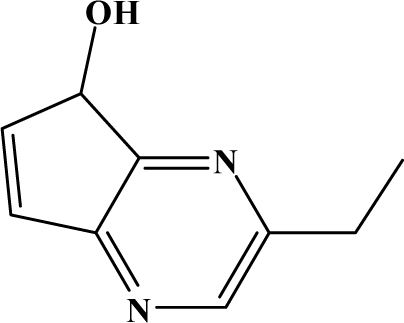	93.8	65.3
II	7e	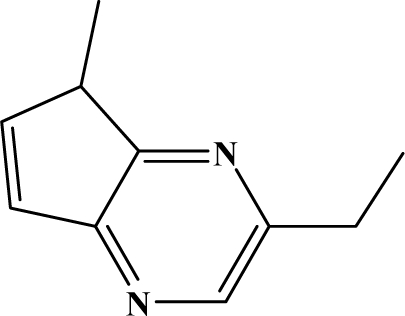	94.9	69.9
II	7f	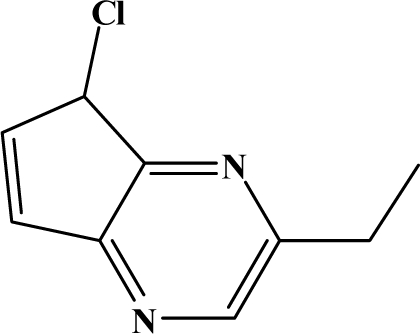	86.0	57.9
II	7g	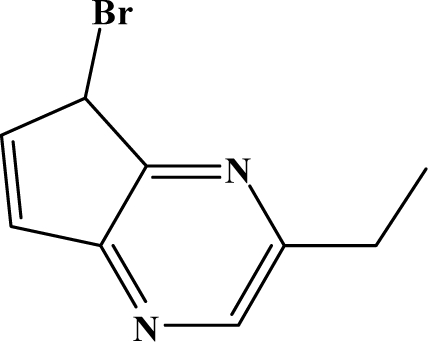	85.3	56.1
II	8a	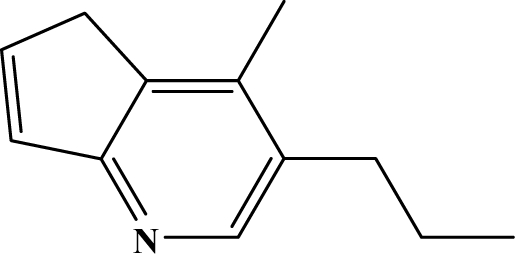	96.3	67.1
II	8b	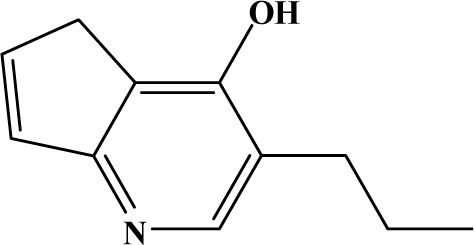	96.2	59.3
II	8c	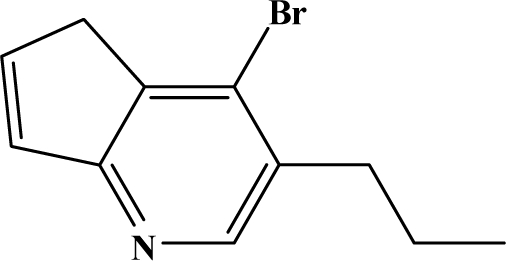	83.9	47.5
II	8d	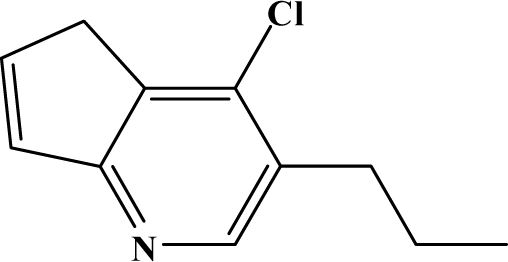	85.7	48.9

a Calculated from Equation (1);

b Calculated from Equation (2).
